# Supplementary material for: Organization and function of Drosophila odorant binding proteins
Source: eLife. 2016 Nov 15;5:e20242. doi: 10.7554/eLife.20242 (PMC5127637; doi:10.7554/eLife.20242)
Supplement: Supplementary file 1. — PAM cleavage sites were located 19-nt downstream of the 5’ end and 37-nt downstream of the 3’ end of the Obp28a coding sequence. DOI: http://dx.doi.org/10.7554/eLife.20242.015 [file elife-20242-supp1.docx]

AATCACAAATGAAACAAAAACCAATGTAGCAAAGTTTTGAGGTTTACAAAACCGTTACTCGTTCGTTAATCGCACTCGTAAACTGTTACTGTTGCCGTGACTGCTACTGTGACTCTCGCTTCACATTTCATACCCATAATGGAAGATAACTAAATTATGATCGATATATGATTTTACATTTAAACACTTTACAAAAATCACTCTGCGAAATAAAGAAGAAAGAGAGGGAACTCTTAACCTTAAGTTTAAGTTGCTTTAAATACTTTTACCTAAACTGTATGTTAATTAATTATGACTTAAGTTTAACCAAACGCAAAAACCACAACATCAATGAATTGATGACTCACGAATAGAATTTGCATGCCGCATTCAATGTATATATATGTAGGAGCGATGTATAATTCTCGATCCACATACGGATCTGTCGAAATTAATTATGACTATTTTATTTTCTAATCGATTACTGAAATACCAATAACCGACAAATGAAAGCCACGCAAATTAAAACCAAGCAAGTGGTATATGGGAAATGAATTCCAAAAGCAAAAGTTACACGCAATCATTAATTTTGTCTTTGAAATCTATCCGATAAGTACCGCGAATAATTGTTTTTGTTGAGATGCATTATCATTTTTATTGTGTAAAGTATAAAAAACCAACAAATATATGCAAAATATATGGAATATATACAAAATATGTTGTTTTAAATGGATTTTCAATGGGAAAAATAATTTTACGCACGATTAGAAAGGTTTTGAAATTTCAAAAATGGATTTTCATATTTTATATGGTTTATGGTCTGCAGGATTGCACATCTGATAGACAATTTTGATACTTATGAATTATGTGTAACGAAGCACATATGCAAGTCATGTTGACATGGGGTTGGGACACTTTTTTAATCACTTAGACCTTTTCTGGGGATGTGCGTGTCTTATGGGGGCGGTCAGCACTTATTGGTCGACCGGCTACATTTTGTTAGCTGATCCATTAGCTGAAAACCCCTAATAACGATCACGATGCGGCCAATTAAGCGACCATTGTAAAGATTGTTAAACGATGTACTATATTATTTCACAGCCGAGCGAGTCACCGATTTAGCTATAAAAGCCTGAGTCTCTCATCAGCATAGACAAGTTCCGTTCAGACACACCGACCTAGCATCATGCAGTCTACTCCAATCATTCTGGTGGCAATCGTCCTTCTCGGCGCCGCACTGGTGCGAGCCTTTGACGAGAAGGAGGCCCTGGCCAAGCTGATGGAGTCAGCCGAGAGCTGCATGCCGGAAGTGGGGGCCACCGATGCCGATCTGCAGGAAATGGTCAAGAAGCAGCCAGCCAGCACATATGCCGGCAAGTGCCTGCGCGCCTGCGTGATGAAGAACATCGGAATTCTGGACGCCAACGGAAAACTGGACACGGAGGCAGGTCACGAGAAGGCCAAGCAGTACACGGGCAACGATCCGGCCAAGCTAAAGATTGCCCTGGAGATCGGCGACACCTGTGCCGCCATCACTGTGCCGGATGATCACTGCGAGGCCGCCGAAGCCTATGGCACTTGCTTCAGGGGCGAGGCCAAGAAACATGGACTCTTGTAATCATTGATGCAGCGCTACCCACCTGGACACGCCGATAAAGTTACCTGGACCACCACACTTGTATCTATAAGTTTTGAATAATCGAGGTCGAGTAAAATAAATGCATTAAAAAAGGATTTGGAAGTCTGCAATTTCTATGTTTAAATTTCTGCGTAGCGGGAAAAATTCAAGACATCACTTTTTTGAAAGCTATTTTTGAAAGTAGCTTAAAAGAGTTTTAAATTAACAAATACAAAAAAGTTTAAAACGCTGAATAGATGGTAAATATGTAAAAGTTTATGGATTTCATAGAAGTTAACAGTTGTATTATTATTTCATACTTTATTATCTCATATTTATTATCTACATTTCCTTTTTAACTAGTTTGAAAAATAACAGTCTTGTGTTGCCTGGCCACACTTTTATACAGTCACTTAACAGAGCCTCTGTTACTGAAAGAAACTGGTTCGAGTCACTGCTAAATCATATCATTTTCCGCAACCGAGGGTAGTTGCTCTGATGGCGGTATTTTGCCACACTTGCAAGTATTTTTTAGTGAATTGTTCTGCTTCATGTTATTATTGCCCCAACAAATTGCGTTCCCCAAAAGTCGGTGGATCTCGTTCGCGTGAGGAGCTCAGCGCTCTGTTAGCGGCAATTCGAAAAGCCAAACTCCACGCCAATACGGTTCGTTCAAATCGCCGCTGCAAACGCACAAACACCTTAAACGCCAAACGCACGTAAATTGCGAGTAATCAACCAGATAATAAAAATGTGTAAACAGCTGTGTGTAGCTCGTTAAAATATATTGTTTATATTCTTAATCGGGATGCTAGAAGCCTGAGGCTGCTAAAAATATTTGCCGTGGGTGTTTGTGTTTTTTTCGCCAAGACCCCCCTCATTCATGCCAAAAGGGGCGTGGGAATTACAGCGTGCGTGTATTGGTGTTTTGTGTATAACATGGATATTCAGGAATTTCGCAGATGAATCCTTTTTACGACAATTTATAGTATTGCCTTAATGCACTTTTGAATTTCTAAATGGAGAGGGGAAAGTAAAGTGAAGAAAAAGTGGTTAGAGCAAGGATAACGTATTAGTCATCGTTGGCAGCGACGTCGACTGCGCAGTCGGCAGCGCATCCTGGCTGCCTGTGCGTGTGTGCGTGCGTGTGTTGGTCTCTCTATTTTTGAATTTCCGCCGGTTTCTGCACAACAACAACAAAAGCAACTGAGCGGGAGAAACAGTGCCCGTGCGACAAAAACAACAACTTGTTTGTTTGATCCCTGAAAGAGAGCACGGAAGAGAGCAGGATGGAGCGAAAGTTTACGCGCGGCTTAAATAAGCTCGGCTCAGCGGTGCAAATGAGAGAGAATGTCTCTCAGCTGGTGCGAGAGAGCGCGGTTTTGGTAAGTTCAGAAATTCCCTTATTACACCTGCTTATTGCCATCTCACTCACTCCCCCTAGACTATTTTTGACTTTCAGAAGTGCAGTTGTTGGTTTTCATACATGTGTATATGC

**Annotated sequence legend:**

Green highlight = PAM sequence

Yellow highlight = Seed sequence

Red highlight = Remainder of seed sequence past Cas9 cut site

Teal highlight = Screening primers

Blue text = Coding sequence

Larger font = Excised portion (replaced by DsRed cassette)

Underlined text = Homology arms
